# The Peer Model Advantage in Infants’ Imitation of Familiar Gestures Performed by Differently Aged Models

**DOI:** 10.3389/fpsyg.2012.00252

**Published:** 2012-07-19

**Authors:** Norbert Zmyj, Gisa Aschersleben, Wolfgang Prinz, Moritz Daum

**Affiliations:** ^1^Ruhr-Universität BochumBochum, Germany; ^2^Saarland UniversitySaarbrücken, Germany; ^3^Max Planck Institute for Human Cognitive and Brain SciencesLeipzig, Germany

**Keywords:** gestures, imitation, infancy, peers, model age

## Abstract

Infants’ imitation of differently aged models has been predominately investigated with object-related actions and so far has lead to mixed evidence. Whereas some studies reported an increased likelihood of imitating peer models in contrast to adult models, other studies reported the opposite pattern of results. In the present study, 14-month-old infants were presented with four familiar gestures (e.g., clapping) that were demonstrated by differently aged televised models (peer, older child, adult). Results revealed that infants were more likely to imitate the peer model than the older child or the adult. This result is discussed with respect to a social function of imitation and the mechanism of imitating familiar behavior.

## The Peer Model Advantage in Infants’ Imitation of Gestures Performed by Differently Aged Models

In the past decades, infants’ imitative abilities have received a widespread interest. The two main lines of research focused on the question of when infants start to imitate and which situational factors and mental states of a model infants take into account in their imitative behavior (e.g., Meltzoff, 1995; Gergely et al., 2002; Jones, 2007).

Recent research has additionally shown that the characteristics of the model (i.e., who performs the target action) has a strong impact on infants’ likelihood to imitate an observed action. First, 14-month-olds are more likely to imitate models who previously demonstrated their reliability than models who showed they were not reliable (Zmyj et al., 2010; Poulin-Dubois et al., 2011). Second, 14-month-olds are more inclined imitating models that belong to their own cultural group as compared to models who belong to another cultural group as indicated by language (Buttelmann et al., in press). Third, a model’s age has been shown to influence infant imitation likelihood (Ryalls et al., 2000; Seehagen and Herbert, 2011; Zmyj et al., 2012). Accordingly, controlling for a model’s characteristics is an important and still underestimated factor with respect to infants’ imitative abilities. Both the influence of the model’s reliability and the model’s cultural group have provided rather clear evidence concerning infants’ respective imitative behavior. In contrast, studies focusing on the role of the model’s age have provided mixed results so far.

Only a few studies have systematically investigated the role of a model’s age on infant imitation, with three of these tapping object-directed imitation (Ryalls et al., 2000; Seehagen and Herbert, 2011; Zmyj et al., 2012). Ryalls et al. (2000) presented 14- to 18-month-old infants with an older real-life child model and a real-life adult model performing three-step actions. These actions could be either novel (e.g., making a rattle) or familiar (e.g., cleaning a table). Infants were more likely to imitate the older child model immediately after demonstration. It was not reported whether there was a difference between novel and the familiar actions. It also remained an open question how same-aged models affect infant imitation since the so-called “peer model” was in fact a 3-year-old child and thus around 1.5 years older than the participants.

Seehagen and Herbert (2011) investigated 15- and 24-month-olds’ imitation of the novel action “making a rattle” that was presented either by a televised 2-year-old or by a televised adult. Infants were tested immediately after the modeling phase or after a 10-min delay. Fifteen-month-olds were more likely to imitate the adult model than the peer model when they were tested immediately after having observed the demonstration. Twenty-four-month-olds, however, imitated the action equally well from both models when tested immediately after demonstration but showed a preference for imitating the peer model after a 10-min delay. In this study, the 2-year-old model was only a genuine peer model for the 2-year-olds and not for the younger participants. It again remains unclear whether the results are based on a development of imitation skills between 15 and 24 months or on the differences in age between imitator and imitate of the younger participants.

The relation between action familiarity and the model’s age in infant imitation was investigated more closely by Zmyj et al. (2012). Fourteen-month-olds observed a same-aged peer, an older child, or an adult model performing novel or familiar actions. The results showed that when these models performed a novel action (i.e., illuminating a touch-sensitive light by making contact with the head), the likelihood of imitating this unusual action increased with increasing age of the model. In contrast, infants were more likely to imitate familiar actions (e.g., putting beads into a cup) from a peer model than from an older child model or an adult model.

The three studies just reported all tapped object-directed imitation. There is only one study that aimed to investigate the role of a model’s age for actions involving no object. Abravanel and DeYong (1997) reported that there were no differences in copying facial expressions in infants between 3 and 6 months of age irrespective of whether the model was a televised adult or an animated cartoon infant resembling a 5-month-old. Since it is unclear whether imitation from a cartoon peer model and a televised adult model can be compared directly and the imitative abilities of infants in the first 6 months of life remain a topic of debate (Meltzoff and Moore, 1994; Jones, 1996; Anisfeld, 2005), only limited conclusions can be drawn from this study. Accordingly, it is an open question how imitation of familiar gestures is affected by a model’s age.

The apparent mixed results from studies investigating the imitation of differently aged models raise questions concerning why infants imitate selectively (the function of imitation) and how action perception modulates action production (the mechanism of imitation). In order to explain the seemingly mixed results, one should consider two potential functions of imitation: the cognitive and the social function (Užgiris, 1981). The cognitive function serves the fast acquisition of novel behavior, even if the causality behind this behavior is opaque (Csibra and Gergely, 2006). In this context, imitation is one learning mechanism amongst others and many researchers assume that human culture builds on the ability to imitate (e.g., Tomasello, 1999). Accordingly, empirical studies on imitative learning highlight the importance of using novel behavior as stimuli (e.g., Meltzoff, 1988). This has led to an ongoing debate about which behavior counts as imitation (for an overview, see Paulus, 2011). Even if social cues such as making eye-contact between imitate and imitator are investigated, this kind of research focuses on how relevant knowledge is transmitted (e.g., Csibra and Gergely, 2006).

In contrast, the social function of imitation serves affiliation with others and non-verbal communication (Užgiris, 1981). Since imitation in this context is not conceived of as a learning mechanism, the target behavior is not designed to be novel (e.g., Nadel, 2002). According to Over and Carpenter (2012) the social function of imitation is subdivided into three domains that are not mutually exclusive. First, by imitating others one can identify with them and make oneself more similar to them. In short, one intends to “be like” them. This social goal is neither necessarily conscious nor communicative. For example, adults imitate the gestures of others even if the models are televised (Lakin and Chartrand, 2003, see McGuigan et al., 2011 for similar results in children when observing irrelevant actions). Second, imitation serves a communicative function by carrying the message “I am like you.” Again, this is part of imitation of gestures in adults (Chartrand and Bargh, 1999), as well as in toddlers: They frequently take turns imitating each other in ways that mirror verbal communicative exchanges (Nadel et al., 1999). This behavior peaks at 3 years of age and then decreases with increasing command of language, underlining the communicative nature of imitation. Third, imitation is exhibited when individuals perceive social pressure to conform with others. This social pressure exists in explicit teaching situations (Schmidt et al., 2011) but is also present when subtle social cues such as eye-contact are involved (Brugger et al., 2007).

If infants are presented with novel actions, a cognitive function of imitation – making sense of and learning a puzzling behavior – predominates the social function of imitation by identifying, communicating, or conforming with the model (although a social function is still possible). Infants gather information from models that are likely to be competent (Zmyj et al., 2010). Since adults can be perceived as more competent than peers when acting on mechanical devices, infants imitate adults more often than peers in that context (Seehagen and Herbert, 2011; Zmyj et al., 2012, Experiment 1). However, in the context of familiar behavior there is, by definition, nothing new to be learnt, so the social function might be predominant in infants’ imitation in such situations. In Zmyj et al. (2012, Experiment 2) study, in which peers predominantly imitated familiar actions from peers than from older models, the models were absent and televised. Hence, it is unlikely that infants communicated with the models or felt the pressure to conform with them when they imitated. We suggest that in this scenario infants’ imitation served an identificatory function: to “be like” the model. Infants identified more strongly with similar others, i.e., with peers, than with less similar others, i.e., with older models, which lead to the “peer model advantage” in infant imitation. Since Ryalls et al. (2000) used two familiar and two novel actions and only reported a mean score, their finding is inconclusive with respect to the involvement of the two potential functions of imitation: cognitive and social.

The identificatory function that may underlie imitation the imitation of familiar behavior performed by televised peer models is related to the much discussed general mechanism of infant imitation. According to Meltzoff and Moore (1997) infants’ action perception and action production are processed in a supramodal way from birth, so that visual and proprioceptive information is coded in the same format. This common framework enables infants to detect equivalences between their own acts and the ones they observe (for related accounts see, Prinz, 1997; Heyes, 2001). Relatedly, Paulus (2011) suggested that the infants’ motor system is more strongly activated if the observed behavior is in their own motor repertoire. Accordingly, the movements of models that are biomechanically more similar to the infants’ movements, that is peers, could elicit more motor resonance, which leads to more imitation. This general mechanism of imitation is principally open for any function of imitation. Meltzoff (2005) especially focused on the identificatory function of imitation and the understanding of internal states of others. He drew a developmental line from imitation to the awareness to “be like” others: If infants connect own and other actions (as demonstrated in infant imitation), and they are able to experience the relationship between own actions and own internal states, they understand others as entities with internal states because they act “like me.” Meltzoff’s account shows how the imitative architecture in humans serves a genuine social goal. It has been further proposed that the social function of imitation evolved as a “social glue” between individuals in human phylogeny (Lakin et al., 2003).

The discussion on the function of peer imitation and the mechanism involved in peer imitation raises the question of how infants imitate familiar gestures performed by differently aged models. So far, only object-directed actions have been used to investigate infant imitation of differently aged models. Testing imitation of familiar gestures in 14-month-olds might be informative for two reasons: First, using object-directed actions as stimuli, as in Zmyj et al. (2012, Experiment 2) study, always involves the possibility that infants are unfamiliar with the demonstrated actions, which could trigger the cognitive function. In fact, the “peer model advantage” in Zmyj et al.’s study could not be found for every action. Using familiar gestures reduces this confound because everyday gestures such as waving or clapping are highly likely to have been observed by the infants in the past. Second, studies on gestural imitation in infancy have shown that infants are less likely to imitate gestures compared to object-directed actions (Christie and Slaughter, 2009), and that they imitate gestures on relatively low levels (Rodgon and Kurdek, 1977; Jones, 2007). This does not necessarily have something to do with a general lack of ability but with a specific lack of similarity between model and infant. This lack of similarity in the context of familiar gestures would lead to a reduced motor resonance and to a reduced identification with the model. Presenting infants with models that are more similar to them, that is peers, might lead to more imitation, as it is the case in adults’ imitation of gestures: Adults imitate televised in-group members (indicated by religion) more likely than televised out-group members (Yabar et al., 2006).

In the present study, we investigated the role of the model’s age in 14-month-olds’ imitation of familiar gestures. At this age, infants are not only capable of imitating different gestures (Jones, 2007), they also differentiate between their imitation of object-directed behavior performed by differently aged models (Ryalls et al., 2000; Seehagen and Herbert, 2011; Zmyj et al., 2012). We hypothesized that when presented with differently aged models demonstrating familiar gestures, infants’ motor resonance would be stronger when they observe similar others, that is peers, and that this would lead to an increase in imitation. This mechanism serves one highly important social function of imitation: The identification with an observed peer.

## Method

### Participants

Participants were thirty-six 14-month-olds (*M* = 13 months; 28 days, range 13;15 to 14;15, 20 girls, and 16 boys). They were recruited from a database of parents who had agreed to participate in infant studies. Five additional 14-month-olds were tested, but not included in the final sample due to fussiness (*n* = 3), interference by the parent (*n* = 1), or lack of interest in the demonstration (*n* = 1).

### Test environment and stimuli

The actions performed by the differently aged models were presented via the software Presentation^®^ on a 24″ television monitor. Each video sequence presented a male model of one of three age groups performing the following four gestures: “*Banging*” (the model raises his hands up to the head and bangs on a table making a corresponding noise), “*Waving*” (the model repeatedly moves his hand up and down), “*Clapping*” (the model claps his hands in front of his body making a clapping noise), “*Hand-to-mouth*” (this action consists of a controlled arm movement toward the mouth, so that the fingertips touch the mouth). These actions have been adapted from a study in which similar aged infants were capable of imitating this kind of behavior (Jones, 2007). A pseudo-randomization of the gestures ensured that the gestures with noise (clapping and banging) and ones that were silent (waving and hand-to-mouth) were presented in an alternating and counterbalanced order. Infants were randomly assigned to one of the three experimental conditions (peer model, older child model, or adult model).

In the three experimental conditions, the model was either a male 14-month-old infant (peer model condition), a 3.5-year-old boy (older child model condition), or a 23-year-old adult male (adult model condition). All models were Caucasians. During the recording session, all models wore identical blue T-shirts and sat at a table in front of white background. The camera settings were identical across conditions. As in real-life, the adult model appeared taller than the older child model. The older child model, in turn, appeared taller than the peer model. The duration of each video sequence was 25 s. Each video sequence presented one of the actions described above. One single action was repeatedly presented by playing it on a continuous loop. Speed and sound of the video sequences were edited to ensure a natural appearance of the repeated actions. Consequently, there was no difference in the rhythm or number of gestures across the three age categories. The model did not talk to the infant and the eyes of the model were not directed toward the camera.

### Procedure

All infants were tested individually with one parent present. The test room was unfurnished and contained only a table, a monitor, and white curtains. During the whole experiment, infants sat on their parent’s lap at a table (80 cm × 60 cm) located between the monitor and the infant. Parents were instructed not to interact or speak with their children during the presentation of the video sequences. The experimenter left the room and started the computer-controlled presentation of the stimulus material. The four video sequences were presented one after the other without any break. A picture of a sun was presented together with a male voice saying “Look, there!” as an attention-getter before each presentation of a video sequence. The distance between the infant and the monitor was ~70 cm. During the presentation of the video sequences, a camera was positioned above the monitor and recorded a close-up view of the infant. Additionally, a second camera was focused on the monitor to record the video sequences demonstrated.

### Data analysis

Infants’ behavior and looking time during the observation of the video sequences were coded from video by a trained observer who was blind to the condition. An action was coded as one of the four target actions if it met the following criteria: banging simultaneously with both hands on the table at least once (*Banging*), moving the fingers up and down with one hand at least once (*Waving*), clapping with both hands at least once (*Clapping*), touching the mouth with the hand at least once (*Hand-to-mouth*). Additionally, infants had to watch either the monitor or their own hands while performing the action. A second independent observer rated 33% of the videos. Interobserver reliability was 0.96 (Cohen’s kappa) for occurrence of the target action. We analyzed whether or not infants performed any of the four gestures whilst watching the four video sequences; each infant received an *imitation score* from 0 to 4 for the number of video sequences in which he or she copied the gestures at least once during their demonstration. In addition, each video sequence served as a *baseline* for the spontaneous production of a body movement. For example, infants’ baseline performance of waving during first video sequence where banging was presented was used as a baseline for the second video sequence where waving was presented. Similarly, the second and third video sequences were used as baseline for the third and fourth video sequences, and the fourth video sequence was used as a baseline for the first video sequence (for an analogous coding scheme see Jones, 2007). Each infant thus received a *baseline score* from 0 to 4 for the number of video sequences in which he or she spontaneously performed the baseline gestures at least once. Infants’ baseline and imitation score, as well as looking time were analyzed parametrically by performing repeated measures ANOVA.

## Results

### Preliminary analyses

Infants watched the demonstration equally closely in all conditions (proportion of looking time toward the display: adult model: *M* = 85.3%, SE = 3.1%; older child model: *M* = 80.0%, SE = 1.9%; peer model: *M* = 85.6%, SE = 3.1%), *F*(2, 33) = 1.33, *p* = 0.28, ANOVA. The percentage of acts imitated was not influenced by infants’ sex (female infants: *M* = 30%, SE = 5.9%, male infants: *M* = 31%, SE = 6.2%), *t*(34) = 0.14, *p* = 0.89.

### Main analyses

**Figure 1:**
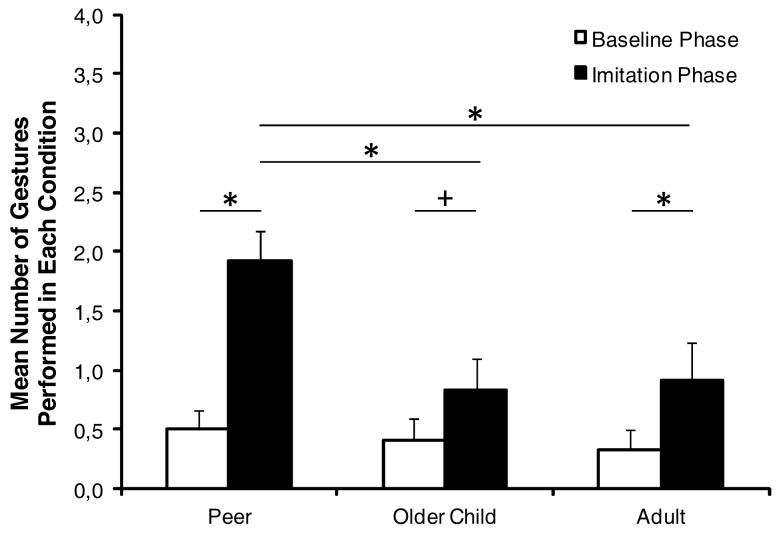
**The mean percentage of gestures the infant performed in each experimental condition**. Asterisks indicate significant differences (*p* < 0.05). Crosses indicate a tendential significant difference (*p* < 0.1).

Figure 1 presents infants’ behavior in the baseline and testing phase across the three conditions. The baseline score was 0.50 (SD = 0.16), 0.42 (SD = 0.17), and 0.33 (SD = 0.17) in the peer model, older child model, and adult model conditions, respectively. In order to test whether infants imitated the models and whether a model’s age affected their behavior, a 2 × 3 (Phase × Condition) repeated measures ANOVA was performed on the imitation scores with Phase (baseline phase and testing phase) as within subjects factor and Condition (peer model, older child model, and adult model) as between subjects factor. This analysis revealed a significant main effect for phase, *F*(1, 33) = 31.15, *p* < 0.001, and a main effect of condition, *F*(2, 33) = 3.79, *p* = 0.03, indicating that infants performed the action that they observed on the monitor above baseline level and that their likelihood of imitating the target movement was different for the three model age groups. These main effects were qualified by a Phase × Condition interaction, *F*(2, 33) = 4.59, *p* = 0.02. Separate analyses revealed that infants imitated in all three conditions, because the target behavior occurred more likely in the testing phase than in the baseline phase (all *p*s < 0.054). During the testing phase infants imitated differently depending on the model’s age, *F*(2, 33) = 5.23, *p* = 0.01. The peer model was imitated more often than the older child model, *t*(22) = 2.89, *p* = 0.01, and more often than the adult model, *t*(22) = 2.72, *p* = 0.01. In contrast, the infants’ imitation did not differ between the older child and the adult, *t*(22) = 0.22, *p* = 0.83. In order to test whether a model’s age influenced infants’ overall activity, baseline scores were additionally compared. There were no differences in the baseline scores, *F*(2, 33) = 0.32, *p* = 0.73.

**Figure 2:**
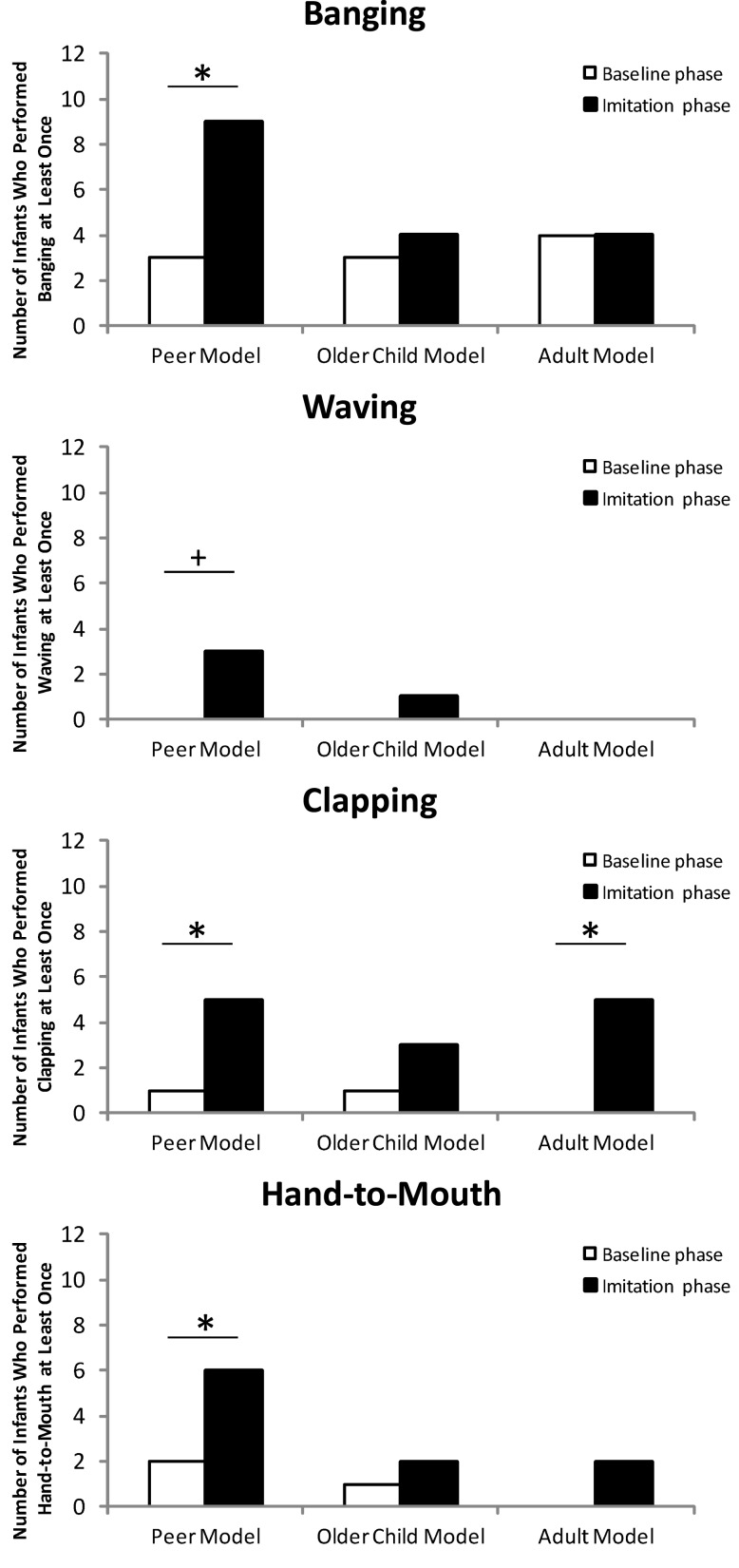
**The number of infants who performed a particular gesture in each experimental condition**. Asterisks indicate significant differences (*p* < 0.05). Crosses indicate a tendential significant difference (*p* < 0.1).

The analyses for each gesture separately did not mirror the analysis of the combined imitation score: There was no difference between conditions (peer model, older child model, and adult model; all *p*s > 0.09, Binomial test). However, there was a significant difference between the baseline phase and the imitation phase for all four gestures in the peer model condition (see Figure 2). In contrast, there was only one significant difference between the baseline phase and the imitation phase for clapping in the adult model condition (*p* < 0.001, Binomial test). All other differences in the older child model and adult model condition were not significant (all *p*s > 0.22, Binomial test).

## Discussion

When presented with familiar gestures performed by a televised model, infants were more likely to imitate peers than older children or adults. In this case, there are no new skills to be learned, and no puzzling behavior has to be made sense of. Moreover, the present experimental setting allowed no communication and no social pressure between models and infants. Imitation in this context might primarily serve a specific social function, namely to identify with the model. Adults (Lakin and Chartrand, 2003) imitate the mannerisms and postures of televised models (see McGuigan et al., 2011 for analogous results in children). This behavior has been interpreted as a means to identify with others and to “be like” them (Over and Carpenter, 2012), and it has been shown that the perceived similarity influences adult imitation of gestures of televised models (Yabar et al., 2006). Infants are also sensitive to the characteristics of model’s that indicate group membership, such as language, and they imitate in-group members more often than out-group members (Buttelmann et al., in press). Identification with similar others might be a general strategy from early on to establish and strengthen group cohesion in different social settings. Since group memberships fundamentally influence the development of a self-concept (Tajfel, 1978), and belonging to a peer group is important in a child’s life (Brown et al., 1986), developmental roots of identification with peers might be already present in infancy.

The tendency to imitate peers has also been observed in interactive contexts as a means of non-verbal communication (Nadel et al., 1999), and as a method to establish social coordination in young children (Eckerman and Whatley, 1977; Eckerman et al., 1989). In the present study we showed that imitating similar others is also present in situations without a communicative context, suggesting that a social function of imitation is present in different contexts as described by Over and Carpenter (2012).

The present study also raises the important question of how imitation is organized in humans. One approach to this question, the principle of Common Coding (Prinz, 1997) proposes that action perception and action production share common representations, suggesting that observing a particular end state of a movement or action effect automatically involves representations that are necessary to execute the same action. In fact, there is evidence that observing the end state of a movement facilitates executing the same movement (e.g., Brass et al., 2000). There is also evidence that this common coding of action perception and action production already exists in infancy (Sommerville and Woodward, 2005; Falck-Ytter et al., 2006; Daum et al., 2011). Different accounts were proposed that mirror this line of thought: Meltzoff and Moore (1997) suggested that executed and observed actions are stored in a supramodal way from birth, so that visual and proprioceptive information are coded in the same format. Along this line, Paulus et al. (2011) introduced the idea of motor resonance in infant imitation: Infants are able to link action perception and action production because they are automatically connected; an observed action that is in the infant’s motor repertoire results in an increased motor resonance and can accordingly be better imitated. Brain imaging studies in adults supported the notion of a close link between action perception and action production of familiar behavior: An observed action activates brain regions differently depending on whether it is in an individual’s motor repertoire (Rizzolatti and Craighero, 2004; Iacoboni, 2005), or not (Brass et al., 2007). This is even the case for one and the same action depending on the individual prior experience (Grush, 2004; Calvo-Merino et al., 2005; for similar results in infants see; Southgate et al., 2010). Due to the age-related differences in biomechanics of models, the gestures demonstrated in the present study were slightly different between model age groups. The gestures performed by the peer model were obviously most similar to the observing infant’s own behavior and might therefore have activated the infant’s action representation more strongly and, accordingly, made an imitative response more likely. The mechanism of imitation and the function of imitation are two rather independent domains. However, in the case of familiar gestures, the general mechanism of imitation (motor resonance) coincides with the social function of imitation in absence of a communicative setting: the identification with similar others. However, since infants imitate novel actions predominantly from adults, referring to the mechanism of imitation which facilitates imitation of similar others cannot explain the full range of studies on selective imitation of differently aged models (Ryalls et al., 2000; Seehagen and Herbert, 2011; Zmyj et al., 2012). Apparently, if infants have the goal to learn (a cognitive function of imitation), other model characteristics such as the perceived competence guide their imitative behavior.

The current finding is also informative in another respect: In a recent study, infants aged between 6 and 15 months watched a real-life adult demonstrate gestures and object-directed actions (Christie and Slaughter, 2009). Whereas most infants imitated at least some of the object-directed actions, only one infant copied one gestural action. Since no procedural details were reported about how gestural imitation was assessed, it is difficult to explain why Christie and Slaughter’s results differ from the present results. One obvious difference between both studies is the age of the participants. They tested infants between 6 and 15 months of age whereas we tested 14-month-old infants which could result in different imitative behavior. The fact that infants in the present study performed more gestures in the baseline phase than infants in the imitation phase in Christie and Slaughter’s study suggests that procedural differences exist and may also account for the difference in results. Additionally, the absence of gestural imitation in Christie and Slaughter may also be attributable to infants’ reduced identification with the adult model. As shown in the present study, infants are less likely to imitate familiar gestures performed by the adult even though they are capable of imitating those gestures, as shown in the peer model condition. Failing to account for infant motivation may lead to a misinterpretation of capacity.

Our study is thus the first to systematically investigate infant imitation of familiar gestures performed by differently aged models. The results showed an increased likelihood of imitating a peer model as compared to older models. This finding might be best explained by infants’ identification with the peer model and the increased motor resonance that is elicited by similar others, i.e., peers. This study also highlights the importance of finding appropriate models when testing infants’ imitation capacities.

## Conflict of Interest Statement

The authors declare that the research was conducted in the absence of any commercial or financial relationships that could be construed as a potential conflict of interest.
